# The “Logic” of Emotion: Do Reasoning Models and Chain-of-Thought Prompting Improve GAI’s Non-Verbal Emotion Recognition?

**DOI:** 10.1192/j.eurpsy.2026.11358

**Published:** 2026-07-31

**Authors:** H. Dery, D. Piterman, Z. Elyoseph, E. Refoua, G. Meinlschmidt, K. Bar, D. Hadar Shoval, A. Geller

**Affiliations:** 1 School of Counseling and Human Development, University of Haifa, Haifa, Israel; 2 Department of Brain Science, Imperial College, London, United Kingdom; 3 Department of Psychology, Bar-Ilan University, Ramat-Gan, Israel; 4 Department of Psychology, Trier University, Trier, Germany; 5 Department of Digital and Blended Psychosomatics and Psychotherapy, University of Basel and University Hospital Basel, Basel, Switzerland; 6 School of Computer Science, Reichman University, Herzliya; 7 Department of Psychology and Educational Counseling, Max Stern Yezreel Valley College, Emek Yezreel; 8 Ruppin Academic Center, Emek Hefer, Israel

## Abstract

**Introduction:**

The assumption that logical reasoning is a general capability that will generalize to non-logical domains is a core tenet in the pursuit of AGI. Non-verbal emotion recognition, a holistic and non-linear task, provides a crucial empirical test for this premise. This study examines whether “reasoning models” and Chain-of-Thought (CoT) prompting, developed for logical tasks, improve emotion recognition or if their efficacy is confined to the symbolic-linguistic domain.

**Objectives:**

This study evaluates whether a designated “reasoning model” outperforms other architectures in non-verbal emotion recognition. It further assesses the effect of Chain-of-Thought (CoT) prompting versus a Zero-Shot (ZS) baseline on accuracy. Finally, it tests if either the reasoning model or CoT prompting can attenuate the previously identified “positivity bias,” a tendency for models to favor positive emotions over negative ones.

**Methods:**

The study employed a 4 (Model) x 2 (Prompting Strategy) factorial design, evaluating four Gemini models (Pro 1.5, Pro 2, Flash 2, and a “Flash Thinking” variant) under both ZS and CoT conditions. The CoT prompt instructed models to apply a structured, psychologically-informed reasoning framework before delivering a final answer. Performance was assessed using the same stimulus set from prior research (Piterman et al., 2025), comprising 36 dynamic bodily gesture clips from the EU-Emotion Stimulus Set (O’Reilly et al., 2016) and 42 vocal prosody recordings from the EU-Emotion Voice Database (Lassalle et al., 2019).

**Results:**

Hypotheses were not consistently supported. The designated “thinking model” did not outperform other models in either bodily gesture or vocal tone recognition; Gemini Pro 2 exhibited the highest accuracy for body gestures. The effect of CoT prompting was not uniformly positive and was highly architecture-dependent: it improved accuracy for advanced models (e.g., Pro 2) but consistently degraded performance for the Flash 2 model across both modalities. The positivity bias was replicated across most conditions. However, a key finding was its significant attenuation in the Gemini Pro 2 model for body gestures. In the vocal tone modality, the bias was also present, but its manifestation was more complex and interacted with the prompting strategy.

**Conclusions:**

Findings challenge the assumption that logical reasoning directly improves non-verbal emotion recognition. Results suggest that imposing analytical processes can disrupt a model’s intuitive, pattern-based capabilities in an architecture-dependent manner. The attenuation of cognitive bias in the most advanced model without explicit reasoning prompts suggests that architectural evolution, rather than prompting techniques, may be the primary pathway toward developing more robust human-like socio-cognitive faculties in artificial intelligence.

**Disclosure of Interest:**

None Declared

